# 16S rRNA metagenome clustering and diversity estimation using locality sensitive hashing

**DOI:** 10.1186/1752-0509-7-S4-S11

**Published:** 2013-10-23

**Authors:** Zeehasham Rasheed, Huzefa Rangwala, Daniel Barbará

**Affiliations:** 1 Department of Computer Science, George Mason University, Fairfax, VA 22030, USA

## Abstract

**Background:**

Advances in biotechnology have changed the manner of characterizing large populations of microbial communities that are ubiquitous across several environments."Metagenome" sequencing involves decoding the DNA of organisms co-existing within ecosystems ranging from ocean, soil and human body. Several researchers are interested in metagenomics because it provides an insight into the complex biodiversity across several environments. Clinicians are using metagenomics to determine the role played by collection of microbial organisms within human body with respect to human health wellness and disease.

**Results:**

We have developed an efficient and scalable, species richness estimation algorithm that uses locality sensitive hashing (LSH). Our algorithm achieves efficiency by approximating the pairwise sequence comparison operations using hashing and also incorporates matching of fixed-length, gapless subsequences criterion to improve the quality of sequence comparisons. We use LSH-based similarity function to cluster similar sequences and make individual groups, called operational taxonomic units (OTUs). We also compute different species diversity/richness metrics by utilizing OTU assignment results to further extend our analysis.

**Conclusion:**

The algorithm is evaluated on synthetic samples and eight targeted 16S rRNA metagenome samples taken from seawater. We compare the performance of our algorithm with several competing diversity estimation algorithms. We show the benefits of our approach with respect to computational runtime and meaningful OTU assignments. We also demonstrate practical significance of the developed algorithm by comparing bacterial diversity and structure across different skin locations.

**Website:**

http://www.cs.gmu.edu/~mlbio/LSH-DIV

## Background

New genomic technologies allow researchers to determine DNA sequences of organisms existing as communities across different environments [1], [2]. The collective sequencing of organisms without culturing and cloning each organism individually is known as "metagenomics". Metagenome samples consist of several DNA sequences originating from all organisms in the examined environment. Through metagenomics, it is possible to study the vast majority of microbes on earth and systematically investigating, classifying, and manipulating the entire genetic material extracted directly from environmental samples. Metagenomics enables scientists to conduct a survey of different microorganisms present in a specific environment, such as water, soil and human body [1,3,4]. By comprehensive study of nucleotide sequence, structure, regulation, and biological functions within the community, the roles played by microbial communities can potentially be examined.

However, sequencing technologies do not provide the whole genome of different co-existing organisms, but produce short contiguous subsequences called *sequence reads *from random positions of the entire genome. One of the grand challenges in the study of metagenome data involves reconstructing the different microbial genomes from a mixture of sequence reads. This is referred to as the metagenome assembly problem. Due to the high species complexity and the short length of sequencing reads from current sequencing technologies, the genome reconstruction goal becomes more difficult to attain if not impossible. Also within a community, microbes (or organisms) vary in abundance, diversity, complexity, genome lengths and may have not been individually sequenced before. Likewise, the current sequencing technologies produce large volume of sequence reads, and reads that may have varying error idiosyncracies [5]. As such,the metagenome assembly problem is complex and challenging [6] and is often subject to further analysis as a collection of short reads.

Targeted metagenome or 16S rRNA gene sequencing has been widely used for the analysis of genetic diversity estimation, enabling deep views into hundreds of complex microbial communities simultaneously. 16S sequences are marker genes, which exist in most microbial species but have variations in their sequences that allow them to be separated into different taxonomical groups [7]. Several metagenome analysis projects use sequencing of 16S genes as a first step in estimating the diversity within a sample. Various computational methods have been developed for the rapid analysis of large sets of reads obtained from targeted metagenome (16S marker genes) or "whole" metagenome studies [5]. Clustering methods have been developed to compare metagenome samples by grouping similar metagenome sequences into bins [8-10]. Other methods use classification techniques to categorize metagenome samples into different phylogenies [11].

In general, two sets of approaches are considered for species diversity estimation in metagenome analysis. The first approach consists of comparative or sequence similarity based methods that rely on homology to separate sequences into different taxonomic levels and classes using annotated database [12-14]. These methods align reads or contigs using global and local sequence alignment algorithms (characters) to identify regions of similarity between sequences [15]. The second approach contains unsupervised clustering methods that identify groups of similar sequences within metagenome samples. This grouping of similar sequences is commonly known as "binning" problem. Different groups in a particular sample is also referred to as Operational Taxonomic Units (OTUs) and the number of OTUs gives an approximation of species diversity in a sample [16-18]. OTU-based approaches are not constrained due to the absence of a complete coverage in taxonomic databases. Several environmental samples contain microbial organisms that have never been laboratory cultured, and as such do not exist in genomic databases.

The OTU assignment can be used to estimate several species diversity estimates such as Chao1 index [19], Shannon diversity index [20] and Abundance-based Coverage Estimator (ACE) index [21]. These OTU assignments and diversity estimates facilitate the process of comparative metagenomics i.e., comparing the genomic content of different community samples. Mothur, DOTUR and ESPRIT are the most widely used methods for OTU estimation [22,23]. QIIME [24] is an open source software package for OTU estimation, taxonomic assignment, statistical analysis and comparison of microbial communities and is primarily used for analyzing high-throughput 16S metagenomic data, generated on a variety of platforms.

One of the purpose of developing clustering methods is to handle the large output of metagenome sequencing projects. We propose a new, scalable metagenomic sequence clustering algorithm (LSH-Div) for targeted metagenome sequences (or called 16S rRNA metagenomes) that utilizes an efficient locality sensitive based hashing (LSH) function [25] to approximate the pairwise sequence operations. LSH algorithm maps the original dimension of input sequences into reduced dimensional space by randomly choosing subset of sequence positions. LSH-based method is also used by Buhler et. al. [26] to detect all pairs of similar segments within long genomes. Our LSH-function is enriched to use gapless, subsequences of fixed length (w-mer), which helps us to reduce the number of false positives and improve cluster quality. The LSH-Div algorithm follows a greedy and iterative framework for assigning OTUs (or clusters) to each sequence. LSH-Div further uses these OTU assignment results and introduces standard species richness metrics which bring more explanation to clustering results.

We assess LSH-Div algorithm on eight environmental samples and one synthetic sample taken from sea water. These samples contain varying complexities of microbial community which give in-depth representation of microbes in sea water[27]. Our evaluation metrics include quality of OTUs, species diversity estimation, computational speedup and pairwise sequence similarity of sequences within each OTU. We demonstrate that LSH-Div is computationally efficient and also produces meaningful and accurate results in comparison to the state-of-the-art OTU estimation algorithms. This work is an extension of our previous work on species diversity estimation [28] and clustering [29].

### Related work

In this section, we briefly explain three state-of-the-art OTU estimation algorithms. Mothur [16] and DOTUR [17] use a PHYLIP-formatted pairwise distance matrix [30] for computing the species richness metrics. In ESPRIT [18], the computation is reduced by using *k*-mer distance between sequences.

MOTHUR and DOTUR are hierarchical clustering approaches that take as input an all-pairwise similarity/distance matrix. The distance matrix calculated by performaing sequence alignment between every pair of sequences within an input metagenome sample. Both approaches support three merge criterion to perform hierarchical clustering given by nearest-neighbor, furthest-neighbor or use of group average. These approaches also use a distance cutoff as an input parameter. This cutoff varaible *d*, defines the maximum allowable distance between sequences within the final cluster (also OTU), such that sequences within a given cluster are at most d% distance apart.

DOTUR reports bio-diversity metrics (e.g., Chao1, Shannon and ACE) with varying distance cutoffs. On the other hand, MOTHUR generates statistics such as count of OTUs, singletons, doubletons and number of sequences in the largest OTU. As a computational expenses, both approaches require memory space for loading the entire all-pairwise distance matrix.

ESPRIT is another sequence clustering algorithm that avoids performing the computationally intensive all-pairwise distance calculation. Instead, the algorithm computes a *k*-mer based distance between pairs of input sequences and achieves computational efficiency. For each sequence, ESPRIT builds a complete genomic alphabet profile of selected *k*-mer and uses that representation to calculate all pairwise distances [18]. In order to reduce further computational complexity, ESPRIT also uses various heuristics. For example, if two sequences are identical or one sequence is a subset of the other sequence, then only longer sequence is kept and the number of occurrence for identical sequences are recorded. This technique reduces the total number of sequences and therefore reduces the time taken by *k*-mer pairwise distance matrix calculation. Using the pairwise input matrix, ESPRIT then performs standard hierarchical clustering.

## Methods

**Figure 1:**
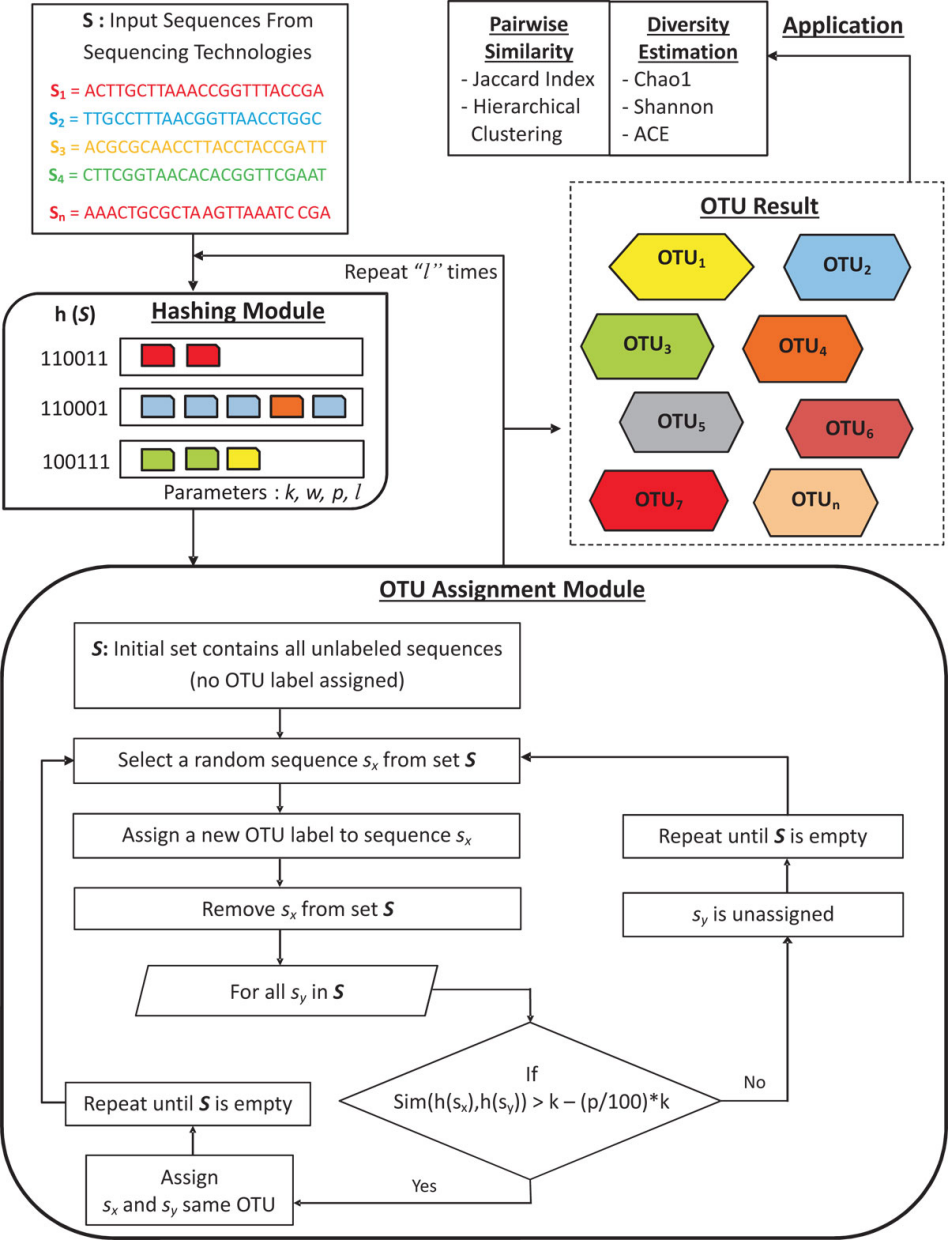
**A complete work flow of LSH-Div algorithm**. A detailed work flow diagram of LSH-Div algorithm for OTU and species diversity estimation. The process goes through different modules such as hashing module, OTU assignment module and results/applications module.

We present our approach to determine species diversity by estimating OTUs from 16S rRNA metagenome sequences using a locality sensitive hashing (LSH) based function. The LSH function reduces the complexity of exact pairwise similarity or sequence alignment. We call our method as LSH-Div (LSH Species Diversity Estimator) and the work flow is described in Figure 1. We first review the basic fundamentals of LSH and then discuss the implementation of the LSH-Div algorithm.

### Locality sensitive hashing

We denote  S as the input set of N sequences. A sequence within  S of length *n *is denoted by *s*. For each *n *length sequence, we choose *k *indices in a random manner. These uniform, random indices are denoted by *i*_1_, . . . , *i_k _*are used to define a hash function for the sequence *s*, given by

(1) $$h\left(s\right)\;=\left\langle s\left[i_{\text{1}}\right],\;s\left[i_{\text{2}}\right],\;.\;.\;.\;,\;s\left[i_{k}\right]\right\rangle$$

The hash function *h*(*s*) in Equation 1 extracts a contiguous *k*-length string from original *n*-length string *s*. The hash function, *h*(*s*) is responsible for transforming the original 4*^n^*-dimensional space to a reduced 4*_k_*-dimensional space. The hash function is "locality-sensitive", as the probability of two strings hashing to the same value varies in direct proportion with their pairwsie similarity [25]. Given two similar strings *s_x _*and *s_y _*which contain at the most *p *different nucleotides, the probability of their hash values being identical is given by:

(2) $$P\left[h\left(s_{x}\right)\;=h\left(s_{y}\right)\right]\geq {\left(1-\frac{p}{n}\right)}^{k}$$

where parameter *p *is the maximum allowable mismatch between the two pairs of strings and *P *[] is the probability which is computed over random choices of the *k *indices, *i*_1_, . . . , *i_k_*.

Naturally, the LSH-based procedure will lead to either false positives (FP) or false negatives (FN) [29]. When the two strings, *s_x _*and *s_y _*are mapped to same hash values even if they are not similar i.e., *h*(*s_x_*) = *h*(*s_y_*), then a FP has occured. This is because of the random sampling procedure that chose only those *k *nucleotide positions that were identical in the two sequences. When the two sequences *s_x _*and *s_y _*are similar but not exactly same, then a FN can occur when the hash function samples those *k *indices where the sequences diverge. This leads to *h*(*s_x_*) ≠ *h*(*s_y_*) even if two sequences are similar in terms of practical purposes. FNs can be substantially reduced by iterating the sampling procedure, multiple times. *l *denotes the parameter for the number of iterations.

**Figure 2:**
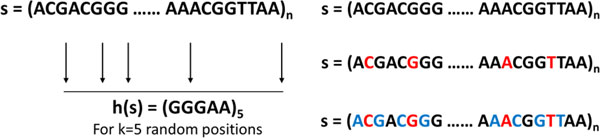
**Selection of k sampled indices along with subsequence length *w***. Construction of randomized hash function using LSH-Div algorithm. *k *uniform, random indices are chosen between 1 *. . . n *to determine hash function h(s). For each index, *w *characters are chosen to the left and right to incorporate gapless subsequence matching.

In order to improve the hashing mechanism, we use a contiguous fixed-length subsequence (*w*-mer), when defining our hash function. Instead, of using a single DNA alphabet at the randomly chosen index *i*, we select the a subsequence around the chosen indices. This is shown in Figure 2. The *w*-mer captures local sequential similarities within the sequences and is used in several bioinformatic applications [31,32]. By comparing a contiguous subsequence rather than single nucleotide at different indices, the method produces more accurate hashing function and reduces FPs.

### Algorithm overview

Figure 1 shows the LSH-Div algorithm procedure.

### LSH-Div parameters

In this section, we describe the parameters of LSH-Div algorithm in detail.

#### Number of sampled indices (k) and subsequence length (w**)**

The hash function provides a mapping from the 4*^n^*-dimensional space into 4*^k^*-dimensional space. Using small values for *k *will lead to small number of partitions, and large number of false positives, since the number of sampled bits or indices will not be enough to disambiguate dissimilar reads.

The use of gapless, subsequence of length *w *enhances the filtering quality as we compare a *w*-mer rather than single nucleotide at a given index position. For pairs of strings to hash to the same key value, requires an exact matching of k * *w *nucleotides. This makes the matching process more stringent and will lead to a reduction in the number of false positives. The selection of *k *sampled indices along with subsequence length *w *is shown in Figure 2.

#### Number of hash functions or iterations (l)

The use of multiple hash functions within the LSH-Div framework reduces the number of false negatives i.e., two sequences that are similar will have a higher chance of being accepted by our filter due to repeated sampling of *k *indices. The results of multiple iterations is combined using a union set operation i.e., a pair of strings are considered to be similar, as long as we see the strings hash to the same values in one of the *l *iterations (or *l *samplings). However, using large values of *l *will lead to dissimilar sequences being mapped to the same hash value and will lead to an increased number of false positives. A good choice of *l *and *k *allows LSH-Div algorithm to produce sensitive partitioning of the sequence data.

#### Percentage of mismatches (p)

In order to allow for pairs of strings that are not exactly identical, but similar (i.e., a few nucleotide mismatches) to pass the LSH-based hamming distance filter, the mismatch factor parameter *p *is implemented as the percentage of allowable mismatches. When *p *is set to 0%, the LSH-based function will consider strings to be equivalent if and only if all the *k *nucleotides or *k w*-mers are exactly identical in both the strings. As an example, when *k *is set to 64 indices and *p *is set to 10% mismatch, then pairs of strings that differ by at most 6 nucleotides or 6 *w*-mers will be considered equivalent. In our current approach, each *w*-mer can be considered a symbol. We do not allow for mismatches within a *w*-mer and is considered as a part of the future directions of this work.

### LSH-Div details

LSH-Div framework consists of different modules such as the hashing module, OTU assignment module and application module. The algorithm begins by first generating LSH functions for all the input sequences in set  S. *h*(*s*) is defined using the parameters of *k *and *w *defined earlier. After computing all the different hash values, the algorithm enters into the OTU assignment module.

The assignment process starts by choosing a random sequence *s_x _*from set  S and assigns the first OTU to that sequence. Then for every other sequence *sy *in set  S, LSH-Div performs hamming distance calculation denoted by *Sim*(,), and computes similarity between using the hash values. Using the *Sim*(,) function we assign every sequence *s_y _*in the cluster (started by *s_x_*) that differs from *s_x _*by a few fraction of nucleotides. Exactly similar strings are be accepted by the hashing function. Sequences, once assigned are removed from the set  S. We repeat the above assiged procedure for assigning OTU labels to all the sequences in the input. To improve the performance of LSH-Div, whole OTU assignment module is repeated *l *times. A new LSH function is created for every iteration. Finally, a union operation is performed on the OTU assignments obtained on each successive iterations.

Previous approaches (discussed above) have are computationally intensive and need a computation of all-pairs of sequence distances for OTU/cluster assignment. In comparison, the LSH-Div algorithm does not require all pairwise distances computation for OTU assignment.

### Computation of pairwise distance cut-offs

An input parameter that is implemented in all diversity estimation approaches includes the distance cutoff parameter. The cutoff parameter provides a maximum allowable distance between all sequences within an OTU. As an example a distance cutoff of 0.05 implies that a sequence within an OTU is at the most 5% distant from every other sequence within the same OTU. For LSH-Div, we first generate the OTU assignments for each sequence. We then compute all pairwise distances (using either the global sequence alignment or *k*-mer distance) between all pairs of sequences within the OTU. We then report the maximum pairwise distance computed, which allows for validation of the distance cutoff parameter. This procedure allows us to compare our approach to MOTHUR and DOTUR, both of which use the alignment distance cutoff and ESPRIT, which uses the *k*-mer distance cutoff.

## Results and discussion

In this section we report on a thorough set of experimetal results. We evaluate LSH-Div on several metrics. Specifically, we assess the performance of LSH-Div algorithm with respect to the number of OTUs, different species diversity metrics and run time. We also perform comparative evaluation.

### Synthetic dataset

**Figure 3:**
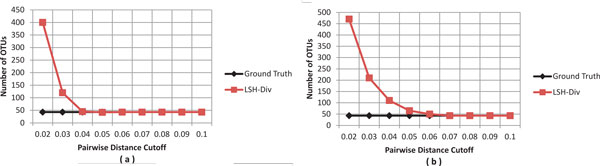
**Analysis of synthetic dataset for number of OTUs**. The graphs show lineage-through-time curves generated by LSH-Div algorithm on synthetic dataset with sequence set containing up to (a) 3% and (b) 5% sequencing errors. The parameter setting used by LSH-Div algorithm to obtain these results is *k *= 30, *w*-mer = 3.

To formalize the accuracy and completeness of the LSH-Div algorithm, we evaluate the performance of our method on a synthetic dataset. We would like to assess whether LSH-Div was able to correctly estimate the numbers of OTUs within an input sample. Since, the synthetic dataset was simulated from 43 species-specific gene sequences, 43 was considered to be the ground truth. Figure 3 shows the number of OTUs at various distance cutoff levels for LSH-Div. We use two synthetic datasets that were simulated to have reads with less than 3% and 5% errors. We observe that LSH-Div efficiently converges towards the ground truth. LSH-Div correctly estimates the number of OTUs (ground truth) at a 4% distance cutoff for the reads that were simulated with a 3% error rate.

### Environmental samples

In Table 1, we present the diversity estimation results for LSH-Div algorithm on the eight environmental samples. We vary the global alignment cutoff distance and show different diversity estimation metrics for cutoffs at 0.03, 0.05 and 0.10.

**Table 1:** Number of OTUs and species diversity estimation by LSH-Div algorithm.

OTUs Cutoff Distance 0.03					
**Sample**	**Reads**	**# OTUs**	**Chao1**	***HI***	**ACE**

53R	11218	1459	3726.53	4.59	3564.58
55R	8680	1461	3915.25	4.92	4274.4
112R	11132	2111	6838.9	5.62	7255.16
115R	13441	1540	3930.08	4.6	3972.27
137	12259	1266	3181.92	4.85	2740.44
138	11554	1306	3031.06	4.61	2998.51
FS312	52569	4321	13942.28	4.78	14345.15
FS396	73657	4594	14228.93	4.18	14826.74
OTUs Cutoff Distance 0.05					

53R	11218	1172	3050.32	4.22	2786.32
55R	8680	1199	3296.38	4.53	3531.21
112R	11132	1795	5781.85	5.19	6126.08
115R	13441	1205	3042.52	4.25	3094.09
137	12259	1041	2595.72	4.60	2317.95
138	11554	1072	2351.90	4.28	2372.84
FS312	52569	3505	10367.35	4.56	10353.72
FS396	73657	3676	10672.02	4.04	10579.55
OTUs Cutoff Distance 0.10					

53R	11218	914	2308.14	3.95	2154.07
55R	8680	963	2418.55	4.35	2538.92
112R	11132	1506	4787.73	4.96	5040.54
115R	13441	943	2446.11	3.83	2409.63
137	12259	817	2028.76	4.24	1831.79
138	11554	845	1985.47	4.02	1827.27
FS312	52569	2771	7038.76	4.22	7444.19
FS396	73657	2876	7534.98	3.84	7370.95

Several species diversity metrics and number of OTUs produced by LSH-Div algorithm on samples taken from Sogin et. al. [27]. The experiment uses three OTU definitions 0.03, 0.05, and 0.10 which define the cutoff levels in distance units. OTU denotes the number of OTUs observed, Chao1 denotes the Chao1 estimate, H´ denotes the Shannon diversity index and ACE denotes the Abundance-based Coverage Estimator. The parameter setting used by LSH-Div algorithm to obtain these results is *k *= 30, *w*-mer = 3.

**Figure 4:**
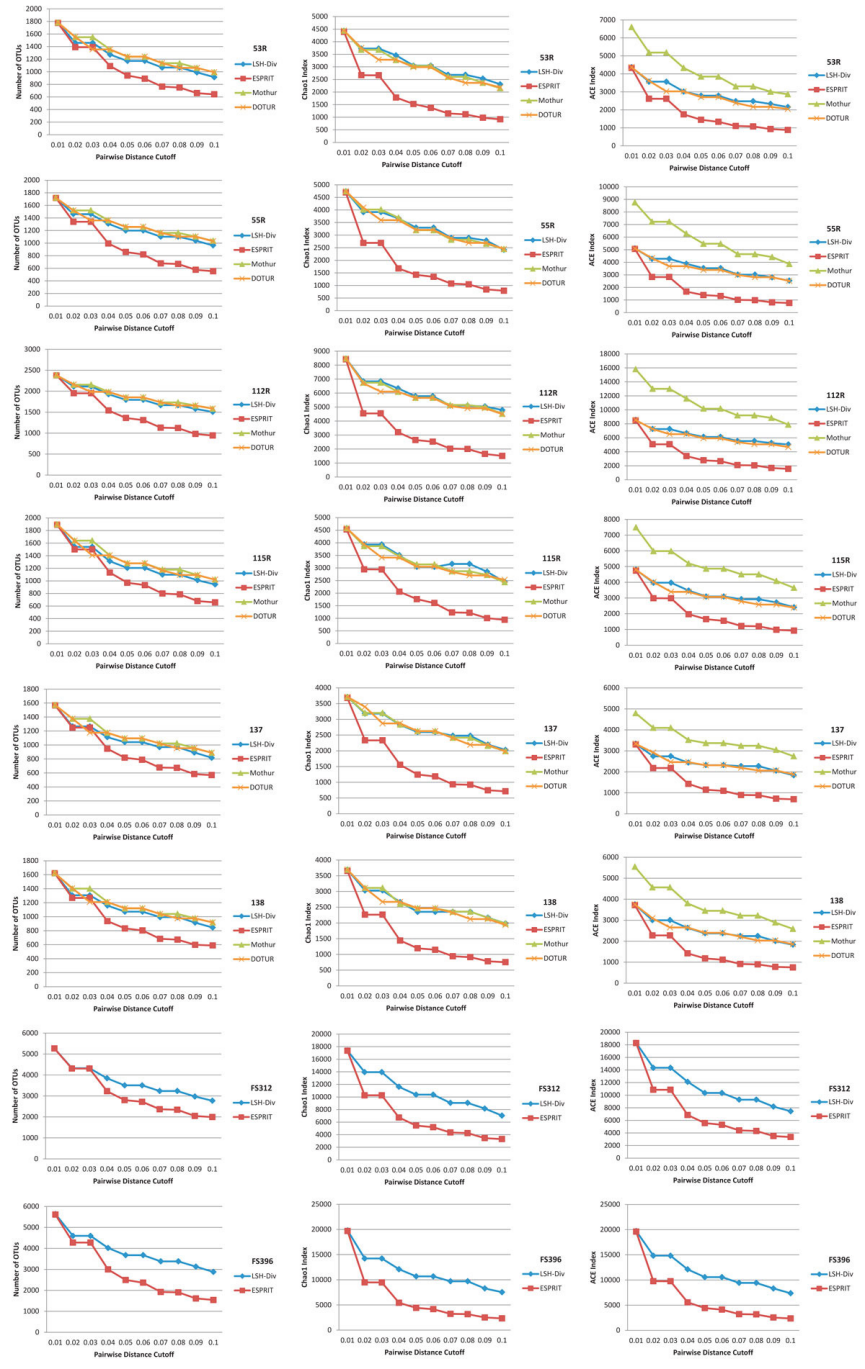
**Species diversity estimation for environmental samples**. LSH-Div algorithm produces different diversity estimates such as Number of OTUs, Chao1 Index and ACE Index. Results are obtained at several distance cut-offs for eight environmental samples. The parameter setting used by LSH-Div algorithm to get these results is *k *= 30, *w*-mer = 3. Mothur and DOTUR are not able to process FS312 and FS319 samples due to large number of sequences and memory limitations.

**Figure 5:**
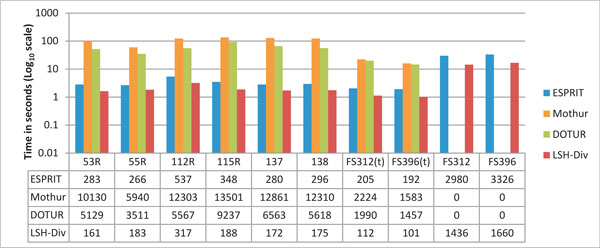
**Runtime comparison**. Computational time taken by LSH-Div, ESPRIT, Mothur and DOTUR to process eight environmental samples in *log*_10 _scale. FS312(t) sample is reduced to 4002 sequences and FS396(t) sample is reduced to 3467 sequences. Avg. Time is reported as average computational time taken by each method across eight environmental samples. Mothur and DOTUR are not able to process FS312 and FS319 samples (runtime is shown as 0) due to large number of sequences and memory limitations. The parameter setting used by LSH-Div algorithm to obtain these results is *k *= 30, *w*-mer = 3.

Figure 4 demonstrates the performance of different methods on number of OTUs, Chao1 index and ACE index for all the environmental samples. All methods show similar estimates except ESPRIT. Mothur shows a higher estimate for the ACE index across all cutoff distances. This indicates that Mothur identifies less number of rare species (*N_rare_*) (see Equations 6, 7 and 8), resulting in high ACE index. ESPRIT, on the other hand, underestimates all species richness metrics. Since, these richness metrics use "singletons" OTUs as numerator and "doubletons" in denominators as shown in Equations 3, 4 and 8, ESPRIT is not able to find the less abundant species within a sample. LSH-Div also provides improved estimates in comparison to Mothur and ESPRIT and is faster in terms of runtime (Figure 5).

### Runtime performance

Figure 5 shows the run times for the different methods on each of the eight samples. As discussed earlier, ESPRIT, Mothur and DOTUR require *O*(*N*^2^) comparisons to compute the pairwise distance matrix, where *N *is the number of sequences in the input set. Mothur and DOTUR use the more expensive Needleman-Wunsch global alignments, whereas ESPRIT uses a *k*-mer based distance function for every sequence pair. ESPRIT also achieves efficiency by removing redundant sequences and also filters sequence pairs that are not very similar. In evaluation of the competing methods, the original environmental samples FS312 and FS396 did not work for ESPRIT, Mothur and DOTUR. These samples required 15GB of memory, and as such were reduced in size by a filtering procedure performed by the ESPRIT method. We report the run time results for the trimmed as well as the original input sequences. We can see a clear performance advantage achieved by LSH-Div in comparison to the other approaches.

### Evaluation of ESPRIT on global alignment pairwise distance

Previous results have shown that ESPRIT underestimates the different diversity metrics. To identify the reason, we setup an experiment to assess the results produced by LSH-Div and ESPRIT using different distance functions for defining the cutoff parameter. Specifically, we implemented the *k*-mer distance cutoff (from ESPRIT) to estimate the OTUs for different inputs varying from 0.01 to 0.10. For the validation purpose, we first compute the maximum pairwise global alignment distance for each OTU produced by ESPRIT and report as the final result, the maximum score obtained across all the different OTU assignments. We also report the validation results by computing the maximum pairwise *k*-mer distance and the global alignment distances for each OTU, produced by LSH-Div. These results are reported in Table 2. We see that ESPRIT fails to meet the global alignment distance-based cutoff criterion whereas LSH-Div satisfies the global aignment distance based cutoff criterion. A similar observation regarding ESPRIT's performance was made in the study by Schloss [33].

**Table 2:** ESPRIT and LSH-Div results for different cut-offs (Sample 112R)

	ESPRIT		LSH-Div	
		
*k*-mer distance	# OTUs	NW	# OTUs	NW / *k*-mer
0.01	2087	0.255	2382	0.0
0.02	1809	0.271	2111	0.014
0.03	1741	0.288	2111	0.014
0.04	1655	0.302	1927	0.032
0.05	1599	0.328	1795	0.048
0.06	1512	0.352	1795	0.048
0.07	1306	0.391	1666	0.065
0.08	1280	0.407	1666	0.065
0.09	1125	0.428	1579	0.081
0.10	1033	0.433	1506	0.098

The results produced by ESPRIT using *k*-mer distance is assessed on Needleman-Wunsch (NW) global alignment distance. The results generated by LSH-Div is evaluated on ESPRIT's generated *k*-mer distance and NW distance. The parameter setting used by LSH-Div algorithm to obtain these results is *k *= 30, *w*-mer = 3.

### Parameter analysis

**Figure 6:**
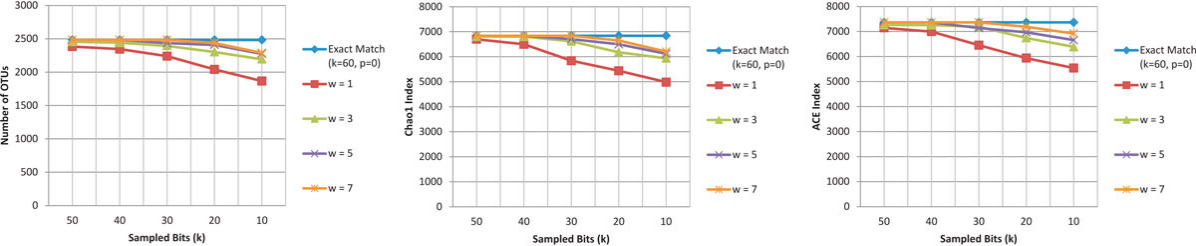
**Effect of varying w-mer and k parameters on LSH-Div algorithm**. Effect on species diversity estimates after varying *w*-mer and *k *parameters (*p *= 0) on LSH-Div algorithm for 112R sample.

We performed an experiment to further reduce the run time of LSH-Div algorithm and maintain the quality of OTU estimates. This was done by reducing the number of sampled indices (*k*), and increasing the *w*-mer size, and thus maintaining the total coverage of the observed sequence. Figure 6 shows the OTU estimates for different parameter settings on 112R sample. Exact Match (*k *= 60, *p *= 0) shows the baseline result when all the sampled bits are used for estimation. We can observe that for several parameter combinations, equivalent OTU quality results are produced.

**Figure 7:**
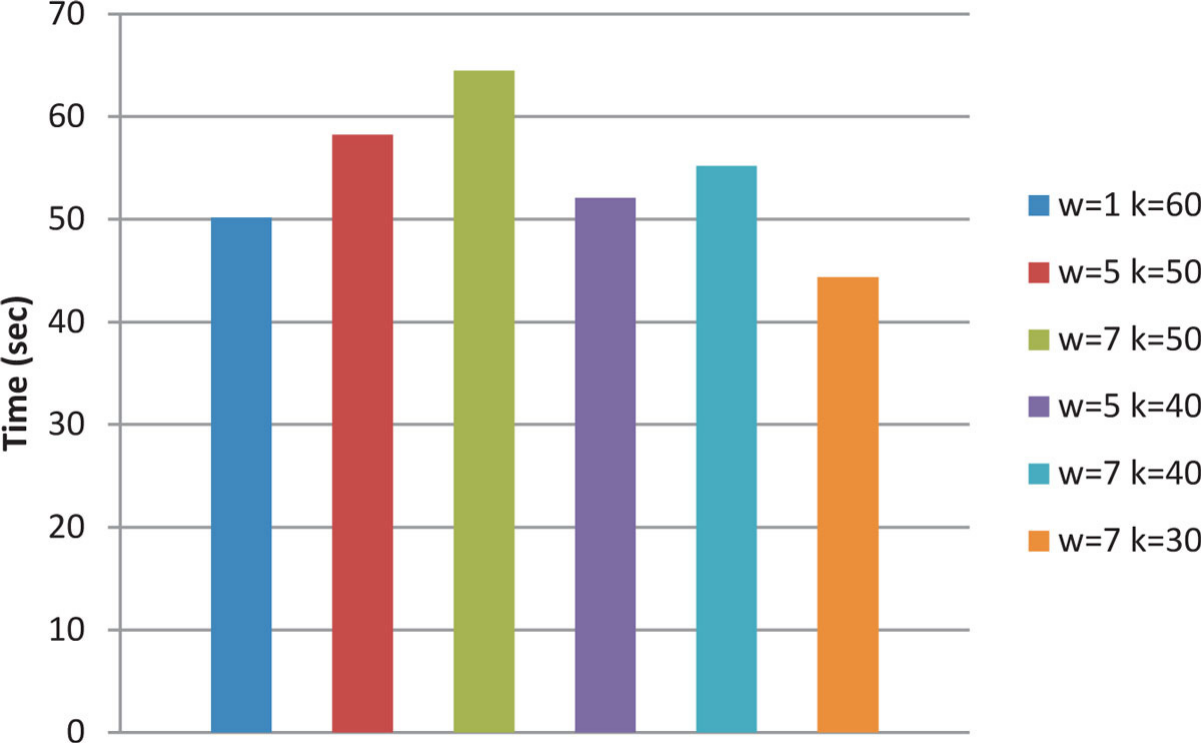
**Run time of LSH-Div algorithm on different parameter settings**. Effect of run time with varying *w*-mer and *k *parameters (p = 0) on LSH-Div algorithm for 112R sample.

We also notice that by using half the sampled indices (*k *= 30) in comparison to exact match (*k *= 60), with *w*-mer size of 7, we produce the same diversity estimates. For these parameter settings, we observe a 1.5 times reduction in the run time. Figure 7 shows the run time of LSH-Div with respect to varying *k *and *w*-mer parameters for sample 112R.

### Significance and impact

Metagenome projects aim to determine the species similarity and diversity across different ecological samples. We use our algorithm to provide an enriched analysis of the variation of microbial species across different skin locations (a real environmental dataset [34]). This dataset contains 112,283 near-full-length 16S rRNA sequences sampled across 21 different skin locations, from ten healthy human controls. These skin sites were selected because they show a predisposition for bacterial infections. The skin sites are categorized as: (i) sebaceous or oily, (ii) moist (typically skin creases) and (iii) dry, flat surfaces. As given by Grice et. al. [34], the sebaceous sites include locations like glabella (between the eyebrows), alar crease (side of the nostril), external auditory canal (inside the ear), retroauricular crease (behind the ear), occiput (back of the scalp), manubrium (upper chest) and back. Moist sites include the nare (inside the nostril), auxiliary vault (armpit), antecubital fossa (inner elbow), interdigital web space (between the middle and ring fingers), inguinal crease (side of the groin), gluteal crease (topmost part of the fold between the buttocks), popliteal fossa (behind the knee), plantar heel (bottom of the heel of the foot), toe web space and umbilicus (navel). Dry sites include the volar forearm (inside of the mid-forearm), hypothenar palm (palm of the hand proximal to the little finger) and buttock. We use the information about skin locations and "genera" as ground truth in our study. The sequence lengths varied from 1280 to 1370 nucelotides (1300 average).

Clustering of metagenome sequences (16S) tends to group sequences that are closely related in the taxonomy/phylogenetic tree (genera) together. Using the LSH-Div algorithm, we cluster all the metagenome sequences. Sequences in this dataset are taken from different skin locations where each skin location is a metagenome sample i.e., genomic sequences of microbes co-existing on the specific skin locations. After binning sequences across all the skin locations, each sequence is associated with one of the OTU labels. Using the OTU label as features for a skin location, we compute the Jaccard index between all pairs of skin locations. Jaccard index measures the proportion of shared clustering labels (species) between the pair of skin locations. A higher index value indicates that the two skin locations are more similar to each other.

**Figure 8:**
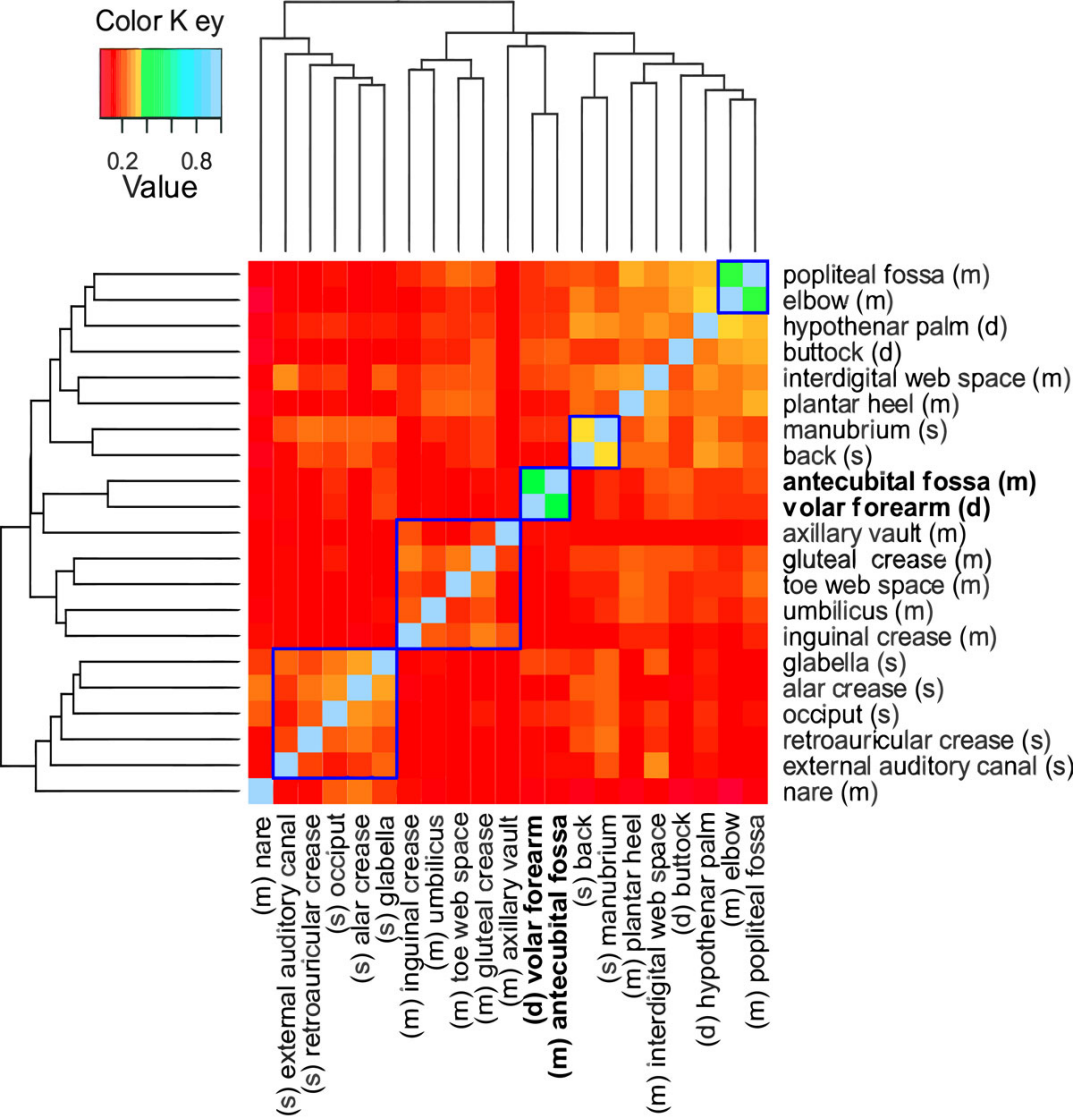
**Pairwise similarity for skin dataset**. Similarity between different skin locations and types using Jaccard Index can be visualized using color key and hierarchical clustering plot. (m), (s) and (d) are defined as moist, sebaceous and dry, respectively. The parameter setting for LSH-Div was *k *= 30, *w*-mer = 3.

Figure 8 shows the pairwise relationship between different skin locations along with the hierarchical clustering results measured using Jaccard index. We can observe that some skin locations which are considered to be similar according to the cluster-membership are not similar in terms of their physical property. For example, the *antecubital fossa *is similar to *volar forearm *according to the Jaccard index but *antecubital fossa *is a moist skin location whereas the *volar forearm *is a dry skin location. An interesting fact about these skin locations is that they both are on the "human forearm". This suggests that microbial content across the skin is determined by the physical property (e.g., dry or moist) and the spatial location (e.g., forearms). This hypothesis was verified by an independent skin microbiome study by Costello et. al. [35], and the hierarchical clustering results produced in Figure 8 was validated by the CROP algorithm [10].

## Conclusion

We developed an efficient and accurate species diversity estimation algorthm referred to as LSH-Div. The key features of our algorithm include the use of a randomized, locality-sensite hashing approach that reduces the complexity of computing expensive global alignments across all pairs of input sequences. The algorithm also incorporates the use of *w*-mers that reduces the number of false positives, and does not increase the run time but leads to better OTU estimation results. LSH-Div was evaluated on synthetic datasets, real world datatsets and after a thorough study, we were able to demontrate that LSH-Div is computationally efficient in comparison to the best OTU estimation algorithms. It also reports the key diversity estimation metrics that are widely used by different biologists. The LSH-Div code is written in Python programming language and is available publicly with the GNU GPL license.

## Materials and implementation

In this section, we describe the datasets and species richness metrics used in the experiments.

### Dataset description

LSH-Div was assessed on both synthetic and real environmental 16S rRNA metagenome samples. The synthetic data was obtained from a previous study conducted in [36]. The synthetic dataset had a total of 345K sequence reads and the reads were generated from forty-three known 16S marker gene sequences. More details about the dataset can be found in [36].

The eight seawater-based metagenome samples were taken from Sogin et. al. [27]. These samples use the 454-based sequencing technology to provide a global in-depth description on the diversity of microbes and their relative abundance in seawater. The description of the samples are given in [27]. The mean length of sequence reads within thesese samples was found to be 60 bp.

We also use a real environmental skin dataset to show the application and significance of LSH-Div algorithm. The dataset covers 21 different skin locations represented by 16S rRNA sequences samples. The total number of sequences is 112,283 with an average length of 1300 bp.

### Species richness estimation metrics

#### Chao1 Index

Chao1 Index [19] is based on the number of OTUs with an individual sequence called "singletons" and the number of OTUs containing a pair of sequences is called "doubletons". The Chao1 estimate is given by:

(3) $$S_{chao1}=S_{obs}+\frac{n_{1}\left(n_{1}-1\right)}{2\left(n_{2}+1\right)},$$

where *S_obs _*is the number of observed species (number of OTUs), *n*_1 _is the number of OTUs with only one sequence and *n*_2 _is the number of OTUs with only two sequences.

#### Shannon Diversity

Shannon Diversity index [20] uses the number of sequences in each OTU and the total number of sequences in the community for calculation.

(4) $$H^{\prime }=-\overset{S_{obs}}{\underset{i=1}{\sum }}\frac{n_{i}}{N}\text{ln}\frac{n_{i}}{N},$$

where *S_obs _*is the number of observed OTUs, *n_i _*= the number of sequences in OTU *i *and *N *is the total number of sequences in the sample. We denote Shannon index as *H*′ in this paper.

#### ACE Index

Abundance-based Coverage Estimator (ACE) Index [21] is based on an "abund" threshold which sets a limit on the number of assigned sequences in an OTU. The number of OTUs with "abund" or fewer sequences are referred to as rare OTUs. The default value for "abund" threshold is set to 10 for every method. The equations for ACE estimate is given by

(5) $$N_{rare}=\overset{abund}{\underset{i=1}{\sum }}in_{i}$$

(6) $$C_{ACE}=1-\frac{n_{1}}{N_{rare}}$$

(7) $$\gamma_{ACE}^{2}=max\left[\frac{S_{rare}}{C_{ACE}}\frac{\overset{abund}{\underset{i=1}{\sum }}i\left(i-1\right)n_{i}}{N_{rare}\left(N_{rare}-1\right)}-1,0\right]$$

(8) $$S_{ACE}=S_{abund}+\frac{S_{rare}}{C_{ACE}}+\frac{n_{1}}{C_{ACE}}\gamma_{ACE}^{2},$$

where *n_i _*is the number of OTUs with i assigned sequences, *S_rare _*is the number of OTUs with 10 or fewer assigned sequences and *S_abund _*is the number of OTUs with more than 10 assigned sequences.

The results produced by LSH-Div can also be used to compute other richness metrics. Also, the rarely occurrent OTUs can be compared against annotated databases in order to identify new species.

### Hardware and software details

The LSH-Div algorithm is available on the supplementary website. It is written using the Python programming language. For experimental evaluation, a single desktop was used. The workstation had 6GB RAM memory witn an Intel-i5 2.53 GHz processor. The competing approaches were all run on the same machine using executables provided by the authors of respective software.

## Competing interests

The authors declare that they have no competing interests.

## Authors' contributions

ZR, HR and DB developed the algorithm details. ZR and HR wrote the code. ZR performed the experimental evaluation. All authors read the manuscript.

## Declarations

The publication costs for this article were funded by NSF Career Award IIS-1252318 awarded to HR.

This article has been published as part of *BMC Systems Biology *Volume 7 Supplement 4, 2013: Selected articles from the IEEE International Conference on Bioinformatics and Biomedicine 2012: Systems Biology. The full contents of the supplement are available online at http://www.biomedcentral.com/bmcsystbiol/supplements/7/S4.
